# Comparative chloroplast genomes and phylogenetic analysis of six *Periploca* species from China provide insights into the distinction of members of this small medicinal genus

**DOI:** 10.3389/fpls.2025.1564539

**Published:** 2025-09-22

**Authors:** Tian Shuai, Qianli Li, Jinlan Long, Xiaoqi Jiang, Linling Wei, Ning Ding, Wei Zhou, Zhikun Wu

**Affiliations:** ^1^ Department of Pharmacy, Guizhou University of Traditional Chinese Medicine, Guiyang, China; ^2^ Department of Pharmacy, Chongqing Duoputai Pharmaceutical Technology Co., Ltd, Chongqing, China; ^3^ Department of Pharmacy, Sinopharm Group Tongjitang (Guizhou) Pharmaceuticals Co., Ltd, Guiyang, China; ^4^ Germplasm Bank of Wild Species & Yunnan Key Laboratory of Crop Wild Relatives Omics, Kunming Institute of Botany, Chinese Academy of Sciences, Kunming, China

**Keywords:** *Periploca*, chloroplast genome, comparative analysis, phenotypic characteristics, codon analysis, molecular markers, molecular identification, phylogenetic analysis

## Abstract

**Introduction:**

The genus *Periploca L*. (Apocynaceae) comprises approximately 17 species worldwide and possesses considerable medicinal value. However, owing to morphological similarities in vegetative organs, specimens and living plants usually lacking flowers and fruits are difficult to determine to species level posing challenges for its proper medicinal utilization.

**Methods:**

In this study, we sequenced and assembled the complete chloroplast (cp) genomes of five *Periploca* species (*P. chrysantha*, *P. forrestii*, *P. calophylla*, *P. floribunda*, and *P. tsiangii*). By combining the new data with the published cp genome of P. sepium and 22 additional Apocynaceae cp genomes from NCBI, we conducted a comparative analysis of all recognized *Periploca* species from China and their closely related taxa to elucidate their phylogenetic relationships.

**Results:**

The cp genomes of *Periploca* exhibit a typical quadripartite structure, with lengths ranging from 153,513 to 154,140 bp and a GC content of 38.1–38.2%. These cp genomes encode 132 genes, including 87 protein-coding genes, 37 tRNA genes, and 8 rRNA genes. Single-nucleotide repeats (A/T) varied among the six species, with forward and palindromic repeats being the dominant long repeat types. We identified 60 long repeat sequences and 55–73 simple sequence repeats, with A/T repeats being the most abundant. Sequence conservation was highest at the SC/IR boundary, while the LSC and SSC regions contained the most highly variable regions. Ten highly variable regions (including *trnK-UUU-rps16*, *rps16-trnQ-UUG-psbK*, *rpoB-trnC-GCA-petN*, *ycf3-trnS-GGA-rps4*, *trnT-UGU-trnL-UAA*, *ndhC-trnC-ACA*, *ycf1-ndhF*, *ndhF-rpl32*, *ndhA*, and *rps15-trnN-GUU*) were identified based on nucleotide diversity and verified by Sanger sequencing, serving as potential molecular markers for the identification of *Periploca* and related genera. Phylogenetic analyses of the complete cp genome sequences successfully distinguished the six *Periploca* species and revealed the evolutionary relationships among them.

**Discussion:**

These findings enrich the genetic resources for *Periploca*, providing insights into molecular identification and phylogeny, and fostering proper medicinal application.

## 1 Introduction

The genus *Periploca* L., traditionally classified within Asclepiadaceae (Jiang and Li, 1977), has been reclassified into Apocynaceae based on the APG IV system (Chase et al., 2016). It comprises approximately 17 species (POWO, 2024), distributed across temperate Asia, southern Europe, and tropical Africa (Jiang and Li, 1977). Most plants in this genus exhibit significant medicinal value. For example, the root of *P. sepium* is commonly used in traditional Chinese medicine as ‘Xiangjia Pi’, which has diuretic effects, dispels edema, alleviates rheumatism, and strengthens muscles and bones (Zhao et al., 2023). *P. forrestii*, known as ‘Heigu Teng’, is one of the top ten Miao medicines in Guizhou, China; the whole plant can be used to alleviate rheumatic bone pain and arthritis, as it reduces swelling and relieves pain (Chen et al., 2021). *P. calophylla*, a medicinal herb called ‘Feixian Teng’ among the Hani and other ethnic minorities in Yunnan, China (Yin et al., 2014), is primarily used to treat lumbago, rheumatism, numbness, bruises, and snake or insect bites (Tan et al., 2010). Additionally, modern pharmacological studies have demonstrated that *Periploca* species exhibit diverse pharmacological effects, including cardiotonic, anti-inflammatory, anticancer, neurotrophic, and cell differentiation-inducing properties (Huang et al., 2019).

Despite its medicinal significance, the taxonomic classification of *Periploca* in China remains challenging. In his monograph on the genus, Browicz (1966) recognized only two species of *Periploca* from China, namely *P. sepium* and *P. calophylla*, and he treated *P. forrestii* and *P. floribunda* as subspecies of *P. calophylla*. In contrast, *Flora Reipublicae Popularis Sinicae* records four species in China: *P. forrestii*, *P. sepium*, *P. calophylla*, and *P. floribunda* (Jiang and Li, 1977), whereas *Flora of China* records five species, adding the new species *P. tsiangii* (Li et al., 1995). A recent review of this genus suggests that there are seven species in China, including two new species, *P. chrysantha* (Yao et al., 2002) and *P. omeiensis* (Zhang et al., 2006). According to the *Duoshi Encyclopedia of Plants* (Duoshi Encyclopedia of Plants, 2024) (https://duocet.ibiodiversity.net/, accessed on 5 October 2024), *Plants of the World Online* (http://www.plantsoftheworldonline.org/, accessed on 15 November 2024), and the *Species 2,000* China Node (Species 2000 China Node, 2024) (http://www.sp2000.org.cn, accessed on 10 October 2024), six species from China are accepted: *P. forrestii*, *P. calophylla*, *P. floribunda*, *P. sepium*, *P. chrysantha*, and *P. tsiangii*, whereas *P. omeiensis* is not accepted by these databases. We contacted the authors of *P. omeiensis* regarding this species; however, they have lost the type specimen. We visited the type locality but did not find *P. omeiensis*; instead, we only found *P. forrestii* there. Therefore, the status of *P. omeiensis* remains uncertain. This taxonomic challenge underscores the urgent need for molecular tools to clarify the species boundaries of *Periploca*.

While chloroplast (cp) genomes data do present certain limitations in species delimitation (e.g., due to hybridization and cytonuclear discordance) (Baldwin et al., 2023), cp genomes have emerged as powerful tools for species identification and phylogenetic analysis owing to their matrilineal inheritance, conserved sequences, and moderate mutation rates (Wu et al., 2024). Advances in next-generation sequencing (NGS) have further enhanced their utility, enabling the use of complete cp genomes as “super-barcodes” to distinguish closely related species (Ji et al., 2021). Successful applications in traditional Chinese medicine, such as in the study of *Polygonatum sibiricum* Redouté, *Artemisia* L., and the Orchidaceae (Cong et al., 2021; Han et al., 2022; Lan et al., 2022), demonstrate the potential of cp genomes for resolving taxonomic ambiguities.

Currently, research on the genus *Periploca* has primarily focused on its chemical composition and pharmacological activities (Fan et al., 2009; Qin, 2018; Huang et al., 2019), whereas studies on its cp genomes and phylogeny remain limited (Long et al., 2022). Due to the morphological similarities in vegetative organs,and the fact that many herbarium specimens and wild living plants lack flowers and fruits, species identification of *Periploca* in China is often challenging. This vegetative similarity complicates the identification process in major herbaria and often leads to the misidentification of plants from other genera as members of *Periploca*. These issues not only hinder the accurate application of *Periploca* in traditional medicine, but also impose constraints on further research.

Therefore, this study aims to: (1) sequence, assemble, and annotate the cp genomes of five *Periploca* species from China (*P. forrestii*, *P. calophylla*, *P. floribunda*, *P. chrysantha*, and *P. tsiangii*), supplementing these data with available *Periploca* sequences from NCBI; (2) analyze their structural features, perform comparative cp genomic analyses, and reconstruct phylogenetic relationships among the accepted *Periploca* species in China; (3) evaluate the utility of cp genomes as potential molecular markers for species identification and taxonomic clarification. By integrating genomic and phylogenetic approaches, this research seeks to resolve long-standing taxonomic uncertainties and provide a robust foundation for the sustainable utilization of *Periploca* in medicinal and conservation applications.

## 2 Materials and methods

### 2.1 Plant materials, principal component analysis, and cluster analysis based on morphological characteristics

**Figure 1:**
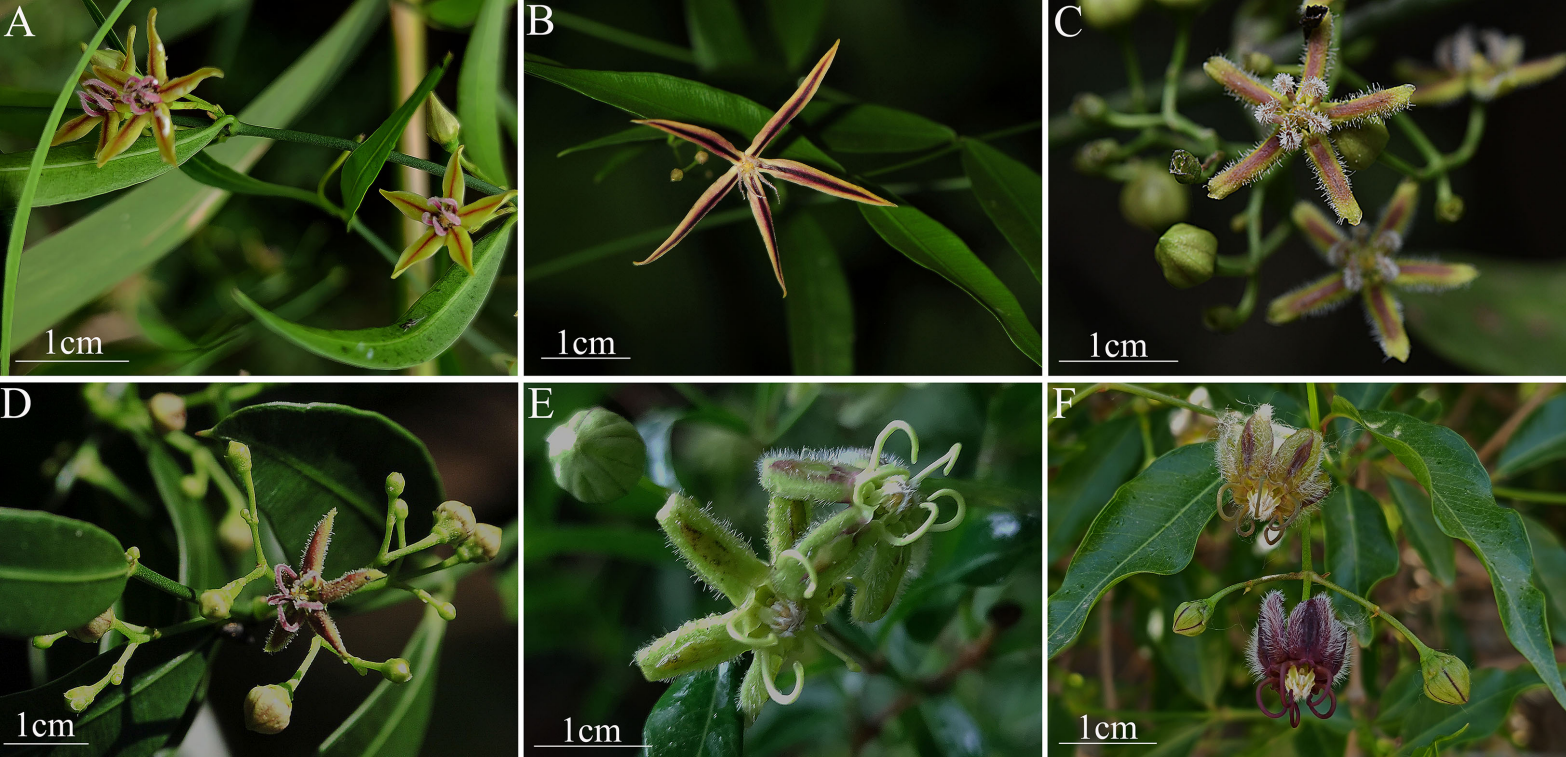
Field morphology of the six *Periploca* species from China. **(A)** *P. forrestii*. **(B)** *P. tsiangii*. **(C)** *P. floribunda*. **(D)** *P. calophylla*. **(E)** *P. chrysantha*. **(F)** *P. sepium*.

Fresh leaves from five *Periploca* species were collected from Xizang, Guizhou, Yunnan, and Gansu, China, during their flowering periods and stored in a –80°C ultra-low temperature freezer. Voucher specimens with flowers were deposited in the Herbarium of Guizhou University of Traditional Chinese Medicine (Cheng-gang Hu, 2274547063@qq.com). The specimens were identified as *Periploca calophylla*, *P. tsiangii*, *P. floribunda*, *P. forrestii*, and *P. chrysantha*, based on floral characteristics (**Table 1**, **Figure 1**). To compare the morphological characteristics of these six *Periploca* species from China, we performed principal component analysis (PCA) and cluster analysis using a total of 90 individuals (15 per species) based on 19 diagnostic morphological traits obtained from observations and measurements of both living plants and specimens, including nine qualitative traits (e.g., leaf arrangement, leaf texture, corolla morphology), and ten quantitative traits (e.g., leaf length, leaf width, calyx diameter) (**Supplementary Table S1**). Qualitative traits were quantified using binary or multistate encoding (0/1/2), whereas quantitative traits were measured repeatedly to obtain mean values (Ryding and Bremer, 1992). All trait data were compiled into a standardized encoding matrix (**Supplementary Table S2**) and subjected to Z-score normalization to eliminate scale differences. PCA was performed using R (R Core Team, 2024), with results visualized using the ‘factoextra’ package (Kassambara and Mundt, 2020) to reduce dimensionality and quantify the contribution rates of key morphological features to species differentiation. For Q-mode cluster analysis, we used IBM SPSS Statistics v26.0 software (IBM Corp, 2019) to assess morphological similarity relationships among the taxa (Zhao, 2020). The analytical parameters were defined as follows: the between-groups linkage algorithm was selected for clustering, and similarity was calculated using squared Euclidean distance. The results were presented as a dendrogram illustrating phenetic relationships among the taxa.

**Table 1:** Sample collection information of the genus *Periploca* from China.

Species	Location	Longitude	Latitude	Altitude	Voucher specimen number
*P. forrestii*	Qianxi,Guizhou,China	106.614E	26.388N	1184 m	HGT201126001
*P. tsiangii*	Libo, Guizhou, China	107.925E	25.286N	722 m	DHGL211003001
*P. floribunda*	Jingdong,Yunnan, China	100.765E	24.280N	1750 m	DHQST201211001
*P. calophylla*	Chayu, Xizang, China	97.276E	28.600N	1889 m	QST190808001
*P. chrysantha*	Maiji,Gansu,China	106.003E	34.352N	1414 m	HHGL230602001

### 2.2 DNA extraction and sequencing

The total genomic DNA was extracted from these samples using a modified CTAB method (Doyle and Doyle, 1987; Fan et al., 2022), and its concentration and quality were evaluated using an ultra-micro spectrophotometer and a blue light transmission imaging system. Subsequently, based on second-generation shallow sequencing using the HiSeq X-Ten sequencing platform, paired-end sequencing with a read length of 2 × 150 bp was performed on the library to obtain raw data. The library construction and sequencing were completed at the Molecular Experiment Platform of the Kunming Institute of Botany, Chinese Academy of Sciences.

### 2.3 Chloroplast genome assembly and annotation

The raw sequencing reads were subjected to rigorous quality control and preprocessing using Fastp v0.23.4 (Chen et al., 2018), which included removal of adapter contaminants, filtering out short reads (< 50 bp), and discarding reads containing > 5% ambiguous bases (Ns). This stringent quality control pipeline yielded high-quality clean reads suitable for cp genome assembly. For the assembly, the cp genome of *P. sepium* (MH752592) was employed as the reference sequence during *de novo* assembly using NOVOPlasty v2.6.2 (Dierckxsens et al., 2017) with the following parameters: genome range: 150,000–190,000; k-mer: 31; combined reads: all clean reads. Gene annotation and analysis were performed using GeSeq v1.84 (Tillich et al., 2017) and CPGAVAS2 v2.5 (Shi et al., 2019). The tRNA genes were initially predicted using tRNAscan-SE v1.21 (Lowe and Eddy, 1997) and were subsequently manually curated in Geneious v9.1.8 (Kearse et al., 2012) to ensure accuracy. The fully annotated cp genomes were then visualized as circular maps using OrganellarGenomeDRAW (OGDRAW) v1.3.1 (Greiner et al., 2019). Finally, the complete annotated cp genome sequences were submitted to the NCBI GenBank database (accession numbers provided in **Supplementary Table S3**).

### 2.4 Repeat sequences and SSR analysis

The forward (F), reverse (R), complement (C), and palindromic (P) repeat sequences in the cp genomes of the six *Periploca* were identified using REPuter v1.0 (Kurtz et al., 2001) with the following parameter settings: a Hamming distance of 3, a maximum repeat size of 60 bp, and a minimum repeat size of 20 bp. Simple sequence repeats (SSRs) were detected using MISA v1.01 (Beier et al., 2017), with the parameters set as follows: the minimum repeat numbers for the mono-, di-, tri-, tetra-, penta-, and hexanucleotides were set to 10, 6, 4, 3, 3, and 3, respectively, and the length between two SSRs was set to 100 bp.

### 2.5 Sequence divergence and nucleotide diversity

A comparative visual analysis of the boundaries of inverted repeat (IR) sequences was conducted on the cp genomes of the six *Periploca* species and *Pentalinon luteum* using CPJSdraw v1.0 (Li et al., 2023). The complete cp genomes of the six *Periploca* species were compared and visualized using mVISTA v2.0 (Frazer et al., 2004) in the Shuffle-LAGAN model, with *P. forrestii* serving as the reference genome. DnaSP v6.12.03 (Rozas et al., 2017) was used to calculate the nucleotide diversity (Pi) values of the large single copy (LSC) region, small single copy (SSC) region, and IR among the six *Periploca* species and to identify divergence hotspot regions within the genome for evolutionary analysis. The step size was set to 200 bp, and the window length was set to 800 bp.

### 2.6 Validation of potential molecular markers

Molecular markers targeting variable regions of the cp genomes were developed to discriminate among the five sequenced *Periploca* species. PCR amplification was performed using 16 primer pairs designed with PRIMER v5.0 (Clarke and Gorley, 2001). Reactions (20 μl total volume) contained: 10 μl Master Mix (Tiangen Biotech, Beijing, China; including 3 mM MgCl_2_, 100 mM KCl, 0.5 mM each dNTP, 20 mM Tris-HCl [pH 8.3], and 0.1 U Taq polymerase), 0.5 μM each primer, 8.5 μl deionized water, and 20–40 ng genomic DNA. The thermal cycling conditions were: 95°C for 3 min; followed by 25–30 cycles of denaturation at 95°C for 30 s, annealing at the primer-specific temperature for 30 s, and extension at 72°C for 30 s; with a final extension at 72°C for 15 min. The PCR products were visualized via 1% agarose gel electrophoresis and subsequently subjected to Sanger sequencing for sequence verification.

### 2.7 Phylogenetic analysis

The phylogenetic relationships between 28 related taxa in Apocynaceae (including the six *Periploca* species from China; **Supplementary Table S3**) were inferred using complete cp genome sequences, with *Halenia elliptica* as the outgroup. The phylogenetic tree was constructed using both maximum likelihood (ML) and Bayesian inference (BI) methods. First, all cp genome sequences were aligned using MAFFT v7.505 with default parameters (Katoh et al., 2002). Then, IQ-TREE v2.2.5 (Nguyen et al., 2015) was used to construct an ML phylogenetic tree under the best-fit model TVM+F+I+R4 selected by ModelFinder of PhyloSuite v1.2.3 software (Zhang et al., 2020). A total of 1,000 bootstrap replicates were used to estimate the statistical reliability of each branch. For the BI analysis, phylogenetic trees were constructed using MrBayes v3.2.5 (Huelsenbeck and Ronquist, 2001) with GTR+F+I+G4 applied as the prior model for nucleotide substitution. Four Markov chains were run for 2 million generations, with the tree sampled every 1,000 generations. After discarding the first 25% of samples as burn-in, the remaining samples were used to construct a consensus tree with posterior probabilities. Finally, the phylogenetic relationships were visualized using the iTOLv7.0 online tool (Letunic and Bork, 2021).

## 3 Results

### 3.1 Principal component analysis and cluster analysis based on morphological characteristics

**Figure 2:**
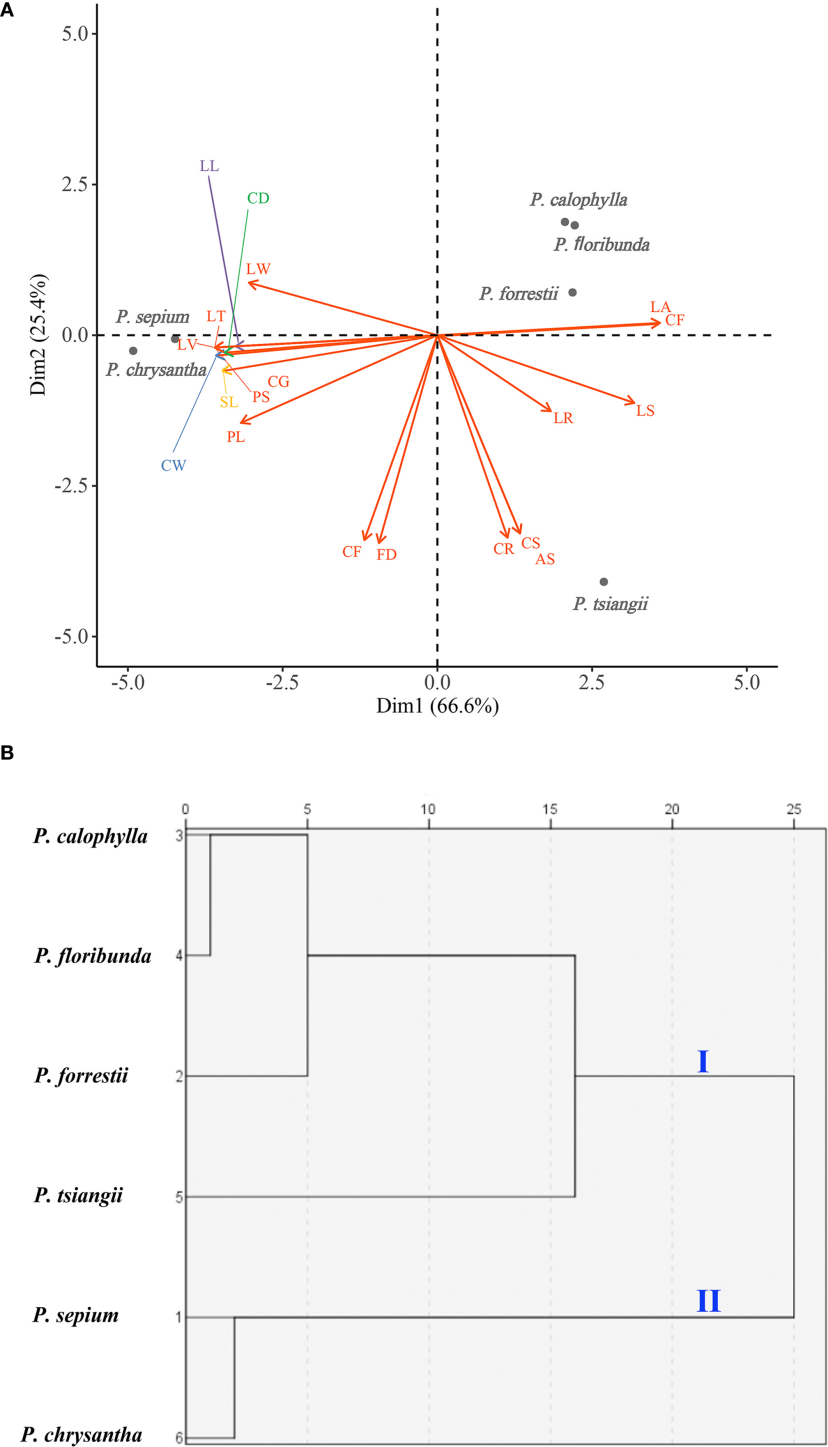
Morphometric analyses of six *Periploca* species from China based on 19 morphological characteristics. **(A)** PCA biplot; (the horizontal axis represents the first principal component (Dim1), while the vertical axis represents the second principal component (Dim2); the length of each arrow corresponds to its contribution rate to the principal components, with longer arrows indicating greater contributions, **Supplementary Table 1** provides the full definitions of the morphological feature abbreviations (e.g., LA) shown in the figure). **(B)** Dendrogram from cluster analysis. The x-axis indicates the Euclidean distance measure.

The PCA biplot results indicated that the cumulative variance contribution rate of the first two principal components (Dim1 and Dim2) reached 92.0%, indicating that these two dimensions effectively explained the morphological variation among the six *Periploca* species. Specifically, Dim1 (accounting for 66.6% of the variance) was significant correlations with 13 morphological traits, including six leaf traits (leaf arrangement (LA), leaf shape (LS), leaf width (LW), leaf length (LL), leaf texture (LT), and leaf venation (LV)) and seven floral traits (corolla morphology (CF), paracorolla indumentum (PS), corolla width (CW), calyx diameter (CD), calyx glands (CG), paracorolla length (PL), and stamen length (SL)). In contrast, Dim2 (explaining 25.4% of the variance) was primarily associated with five floral traits (corolla length (CL), flower diameter (FD), corolla length-to-width ratio (CR), corolla shape (CS), and anther shape (AS)), three of which (CL, CR, and CS) were corolla-related morphological features (**Figure 2A**; **Supplementary Table S1**). In the PCA ordination space, *P. sepium* and *P. chrysantha* exhibited distinct clustering patterns along Dim1, suggesting shared morphological characteristics. Similarly, *P. forrestii*, *P. calophylla*, and *P. floribunda* displayed overlapping distributions, whereas *P. tsiangii* was clearly separated from the other species, reflecting its unique morphological floral traits (linear-lanceolate and markedly elongated corolla lobes) (**Figure 1**). Given the high variance contribution rate of Dim1, traits strongly associated with this axis (e.g., leaf arrangement, corolla morphology, leaf texture, leaf venation, paracorolla indumentum, calyx diameter, and calyx gland number) are proposed as key taxonomic criteria for *Periploca* species (**Figure 2A**).

The clustering analysis demonstrated that at a Euclidean distance of 20, the six *Periploca* species were segregated into two distinct clusters (**Figure 2B**): *P. sepium* and *P. chrysantha* comprised the first cluster, which was distinguished by a decussate leaf arrangement, significantly larger leaf dimensions, membranous leaves, a moderate leaf vein count (20–25) with a relatively sparse arrangement, and each sepal with two glands at the base of its adaxial side, distinctly clavate-thickened and reflexed corolla lobes, and glabrous coronas. The second cluster included *P. calophylla*, *P. floribunda*, *P. forrestii*, and *P. tsiangii*, which displayed a distichous leaf arrangement, a coriaceous or subcoriaceous texture, a notably higher leaf vein density (> 25 veins), comparatively smaller calyx diameters and each sepal with one gland at the base of its adaxial side, uniformly non-thickened, non-reflexed corolla lobes, and densely pubescent coronas. When the Euclidean distance was decreased to 10, the species were further resolved into three clusters (**Figure 2B**): the first retained *P. sepium* and *P. chrysantha*; the second encompassed *P. calophylla*, *P. floribunda*, and *P. forrestii*; and the third was represented exclusively by *P. tsiangii.*

### 3.2 General characteristics of the chloroplast genomes

Combining the published cp genomes of *P. sepium* from NCBI, the cp genomes of the six *Periploca* species from China all exhibited a typical circular quadripartite structure. The total length of the cp genomes in this genus ranged from 153,513 (*P. sepium)* to 154,140 bp *(P. forrestii*), including a LSC of 83,818 bp (*P. sepium*) to 84,941 bp (*P. forrestii*), a SSC of 17,619 bp (*P. forrestii*) to 18,084 bp (*P. chrysantha*) and two inverted repeats (IRa and IRb) of 25,790 bp (*P. forrestii*) to 25,809 bp (*P. sepium*) (**Table 2**; **Supplementary Figure S1**). The overall GC content was relatively consistent, ranging from 38.10% to 38.19%. However, the GC content across the regions was uneven. Specifically, the IR regions had a higher GC content (43.41–43.45%) than the LSC (36.23–36.35%) and SSC (31.92–32.15%) regions. The cp genomes encoded 132 genes in total, including 87 protein-coding genes, 37 tRNA genes, and 8 rRNA genes (**Table 2**). The total lengths of protein-coding genes, tRNA genes, and rRNA genes were 79,677–79,728 bp, 2,882–2,883 bp, and 9,048–9,058 bp, respectively. Among these, 16 genes contained one intron (*atpF*, *ndhA*, *ndhB*, *petB*, *petD*, *rpl2*, *rpoC1*, *rps12*, *rps16*, *trnA-UGC*, *trnC-ACA*, *trnG-UCC*, *trnI-GAU*, *trnK-UUU*, *trnL-UAA*, and *trnV-UAC*), while two genes (*ycf3* and *clpP1*) contained two introns (**Supplementary Table S4**). The *rps12* gene was trans-spliced, with its 5’ end located in the LSC region and its 3’ end in the IR region.

**Table 2:** Characteristics of the chloroplast genomes of six *Periploca* species from China.

Species	*P. forrestii*	*P. tsiangii*	*P. floribunda*	*P. calophylla*	*P. chrysantha*	*P. sepium*
Genome size (bp)	154,140	153,863	153,779	153,717	153,535	153,513
GC (%)	38.19	38.18	38.19	38.19	38.10	38.11
LSC size (bp)	84,941	84,518	84,458	84,364	83,837	83,818
GC in LSC (%)	36.32	36.33	36.35	36.35	36.23	36.25
SSC size (bp)	17,619	17,759	17,735	17,771	18,084	18,077
GC in SSC (%)	32.15	31.94	32.01	31.94	31.92	31.93
IR size (bp)	25,790	25,793	25,793	25,791	25,807	25,809
GC in IR (%)	43.44	43.45	43.45	43.45	43.41	43.43
1st position GC (%)	46.09	46.09	46.09	46.11	46.04	46.06
2nd position GC (%)	38.31	38.35	38.34	38.33	38.24	38.25
3rd position GC (%)	31.10	31.10	31.11	31.09	31.05	31.01
Number of CDS	87	87	87	87	87	87
Length of CDS(bp)	79,728	79,677	79,677	79,677	79,719	79,710
Number of tRNA	37	37	37	37	37	37
Length of tRNA (bp)	2,882	2,882	2,882	2,882	2,883	2,883
Number of rRNA	8	8	8	8	8	8
Length of rRNA (bp)	9,058	9,048	9,048	9,048	9,058	9,058

### 3.3 Repeat sequence and SSR analysis

**Figure 3:**
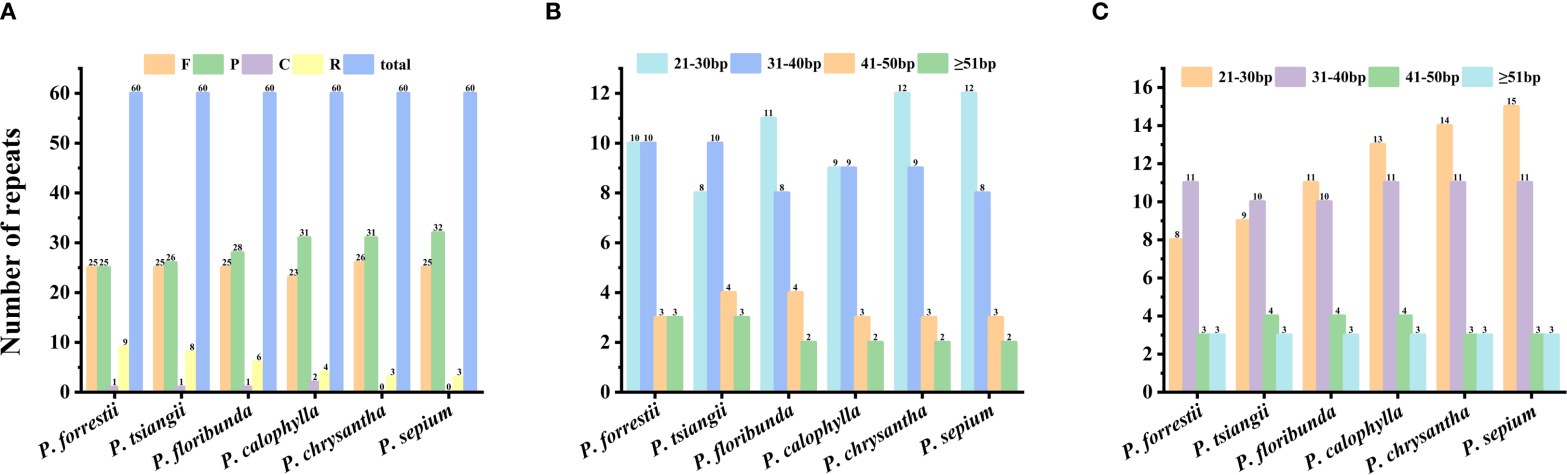
Long repeat sequences of the six *Periploca* species from China. **(A)** long repeat sequence. **(B)** forward repeat. **(C)** palindromic repeat.

We detected four types of long repeats—including palindromic (P), forward (F), complementary (C), and reverse (R) elements—in the six *Periploca* cp genomes (**Figure 3A**). The total number of long repeat sequences across the cp genomes was 60. Among these six cp genomes, all four types were present in *P. forrestii*, *P. calophylla*, *P. floribunda*, and *P. tsiangii*, while only three types of repeats (F, P, and R) were detected in *P. sepium* and *P. chrysantha*. Forward repeats (n = 23–26) and palindromic repeats (n = 25–31) were the most abundant, with their lengths primarily concentrated in the range of 21 to 40 bp (**Figures 3B, C**).

**Figure 4:**
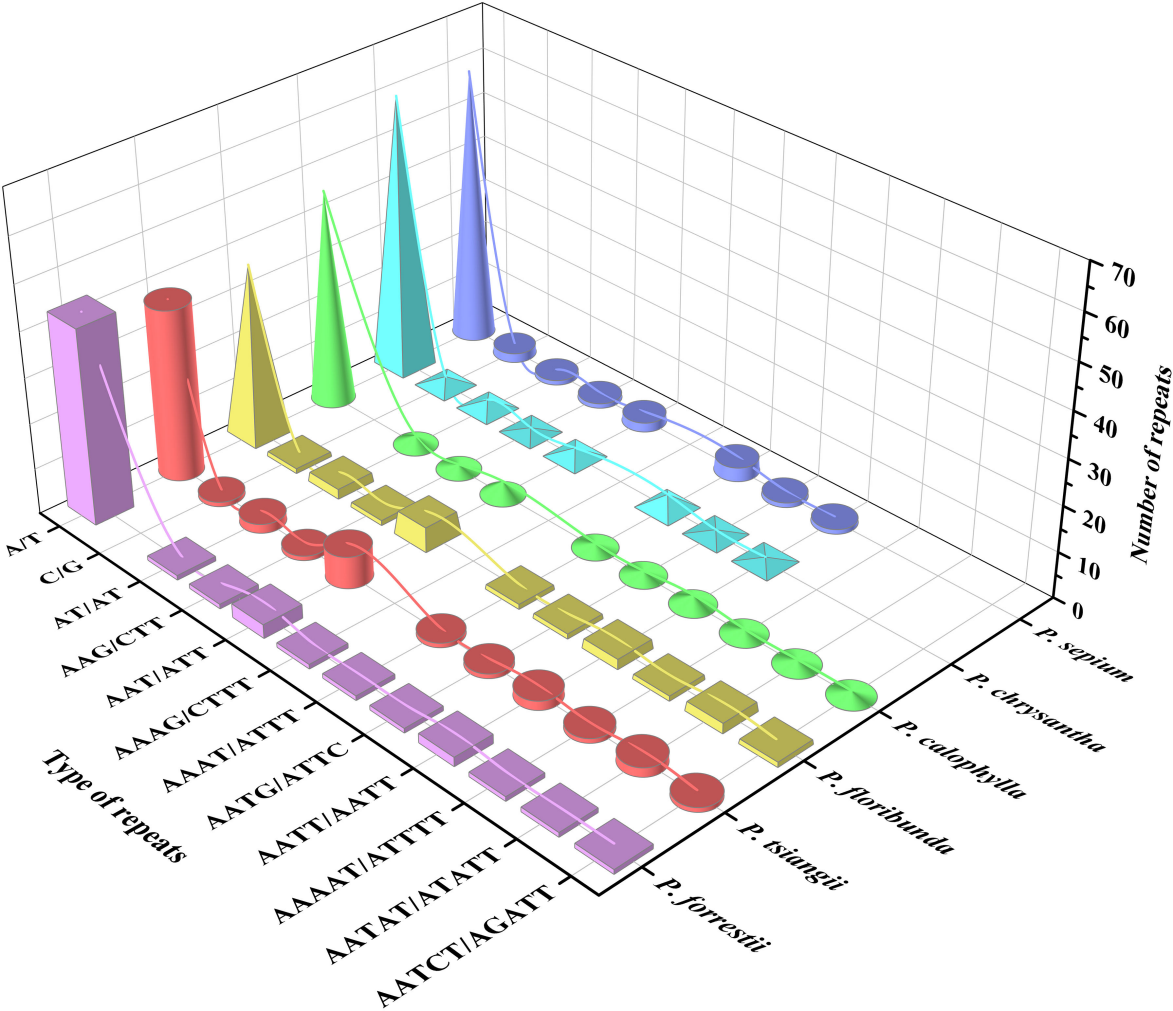
Simple sequence repeats of the six *Periploca* species from China.

A total of 55 to 73 SSRs were detected in the cp genome of the six *Periploca* species, and five types of SSRs (mono-, di-, tri-, tetra-, and pentanucleotide repeats) were identified (**Supplementary Table S5**). The lowest number of SSRs (five) was found in *P. tsiangii* and *P. floribunda*, whereas the highest number (ten) was observed in *P. chrysantha*; six SSRs were detected in *P. forrestii* and *P. calophylla*, and nine in *P. sepium*. All *Periploca* samples from China contained single-nucleotide, dinucleotide, trinucleotide, and tetranucleotide SSRs; however, hexanucleotide SSRs were absent. Additionally, *P. sepium* and *P. chrysantha* lacked pentanucleotide SSRs. Mononucleotide repeats (A/T SSR loci) were the most abundant, accounting for 66.67–84.51% of the total. Except for *P. forrestii* and *P. calophylla*, the remaining four *Periploca* species contained C/G mononucleotide SSRs (**Supplementary Table S6**). The AAAG/CTTT tetranucleotide repeat was exclusive to *P. forrestii* (**Figure 4**).

### 3.4 Expansion and contraction of IRs and nucleotide diversity

**Figure 5:**
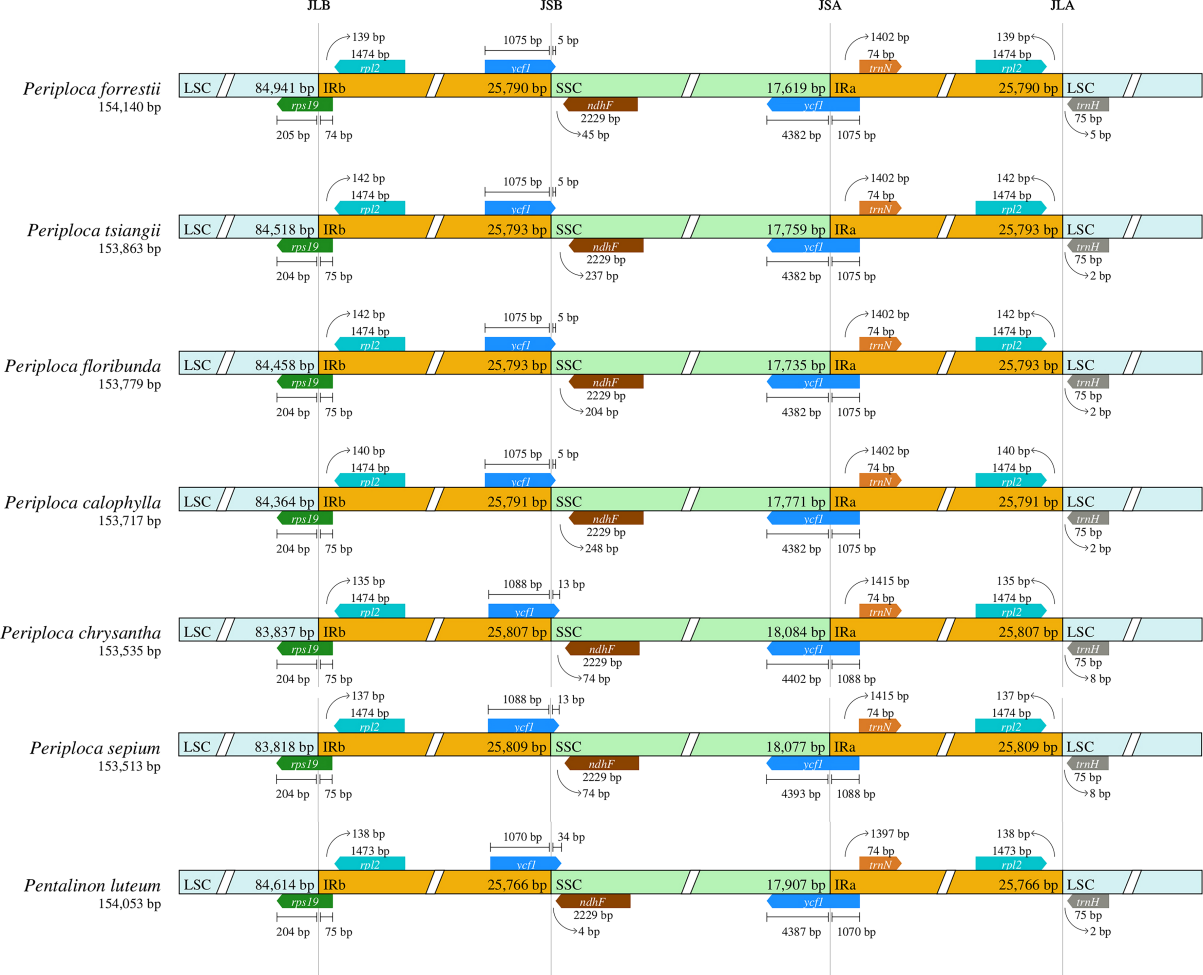
Comparison of the LSC, SSC and IR junction among the six *Periploca* species from China and *P. luteum*. JLB, junction of LSC/IRb; JSB, junction of IRb/SSC; JSA, junction of SSC/IRa; JLA, junction of IRa/LSC.

To elucidate the differences in the cp genomes among the six *Periploca* species from China, a comparative analysis of the IR boundary was conducted between their cp genomes and that of *Pentalinon luteum* (L.) B.F.Hansen & Wunderlin. The results showed that the length of the IR region in the cp genomes of the *Periploca* species ranged from 25,790 to 25,809 bp, whereas that of *P. luteum* was 25,766 bp. The JLB (LSC/IRb) boundaries of the six *Periploca* species and *P. luteum* were all located in the *rps19* gene, with the IRb regions of the six *Periploca* species spanned either 74 bp or 75 bp, showing minimal variation. The JSA (IRa/SSC) boundary and the JSB (SSC/IRb) boundary of the *Periploca* species and *P. luteum* were all located in the *ycf1* gene; however, for *P. sepium* and *P. chrysantha*, the *ycf1* gene extended 1,088 bp into both the IRa and IRb regions; whereas in *P. forrestii*, *P. calophylla*, *P. floribunda*, and *P. tsiangii*, it spanned 1,075 bp into both regions. In *P. luteum*, the *ycf1* gene covered 1,070 bp in both the IRb and IRa regions. Additionally, the seven cp genomes examined contained the *ndhF* and *trnH* genes, which were located exclusively in the SSC and LSC regions, respectively. In contrast, the *rpl2* gene was consistently found within the IR region (**Figure 5**).

**Figure 6:**
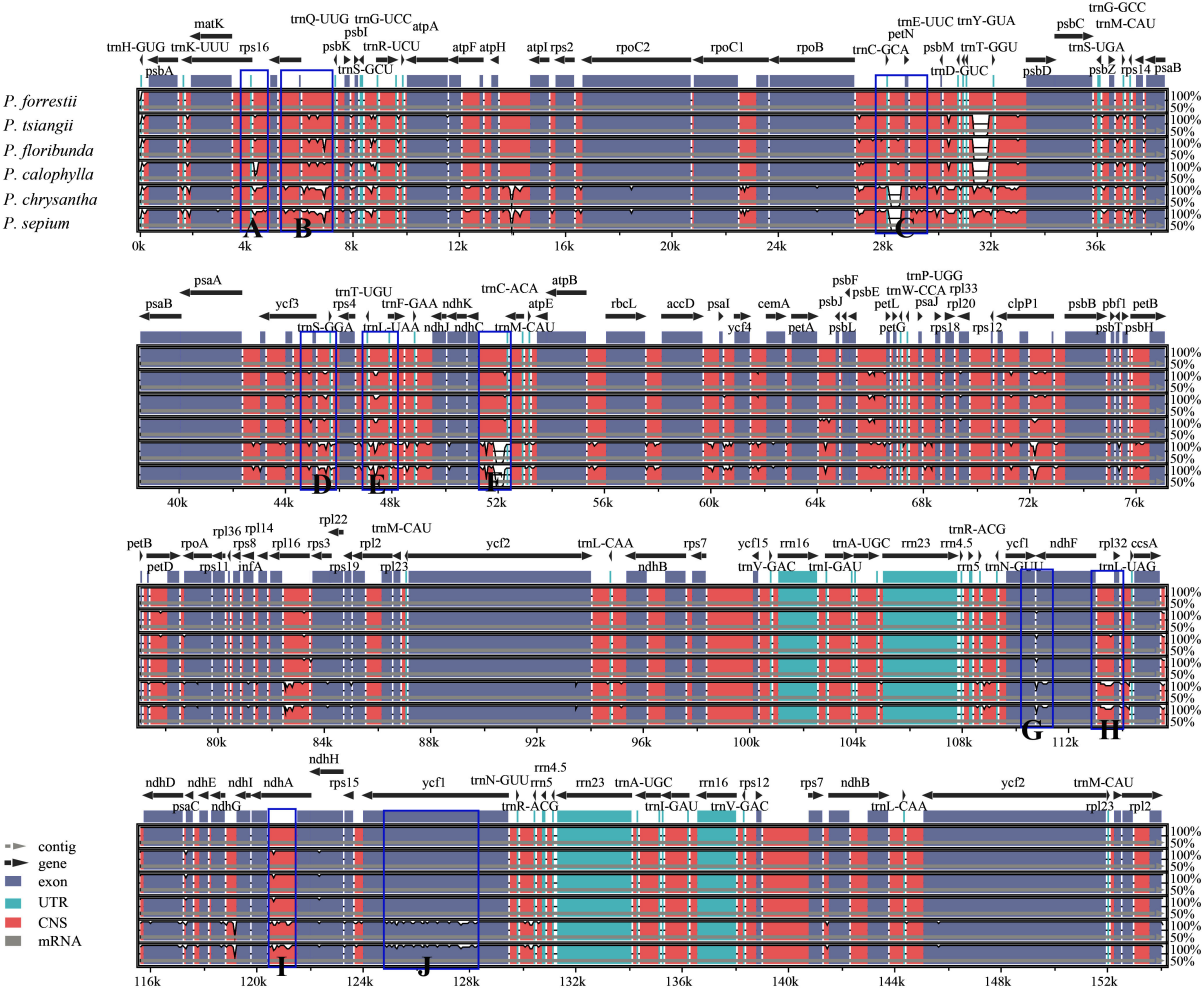
mVISTA map of the chloroplast genome of the six *Periploca* species from China. The x-axis represents the coordinates of the chloroplast genome. The y-axis indicates the average percentage of identity within 50-100%. The direction of gene transcription is indicated by gray arrows, and the genomic regions are color coded for exons, UTR, conserved non-coding sequences and mRNA. The blue frame indicates the highly polymorphic region, with markers **(A–J)** representing distinct intergenic spacer regions (IGS) or protein-coding genes as follows: **(A)** *trnK-UUU-rps16*; **(B)** *rps16-trnQ-UUG-psbK*; **(C)** *rpoB-trnC-GCA-petN*; **(D)** *ycf3-trnS-GGA-rps4*; **(E)** *trnT-UGU-trnL-UAA*; **(F)** *ndhC-trnC-ACA*; & **(G)** *ycf1-ndhF*; **(H)** *ndhF-rpl32*; **(I)** *ndhA*; **(J)** *rps15-trnN-GUU*.

A comprehensive comparative genomic analysis was performed using mVISTA, with the *P. forrestii* cp genome serving as the reference to evaluate sequence divergence among six *Periploca* species (**Figure 6**). The analysis revealed a highly conserved sequence architecture across *Periploca* cp genomes, particularly in the coding regions, which exhibited exceptional stability. In contrast, the non-coding sequences displayed substantially higher variability than coding regions, with mutation hotspots concentrated in the intergenic spacers (IGS). Notably, among the 14 identified highly polymorphic regions, 12 were localized to IGS, including *trnK-UUU-rps16*, *rps16-trnQ-UUG-psbK*, *psbK-trnG-UCC*, *rpoB-trnC-GCA-petN*, *trnT-GGU-psbD*, *ycf3-trnS-GGA-rps4*, *trnT-UGU-trnL-UAA*, *ndhC-trnC-ACA*, *petA-psbJ*, *ycf1-ndhF*, *ndhF-rpl32*, and *rps15-trnN-GUU*. These findings suggest that these IGS regions may experience accelerated nucleotide substitution rates at the species level, rendering them promising molecular markers for future phylogenetic analyses within the *Periploca* genus.

**Figure 7:**
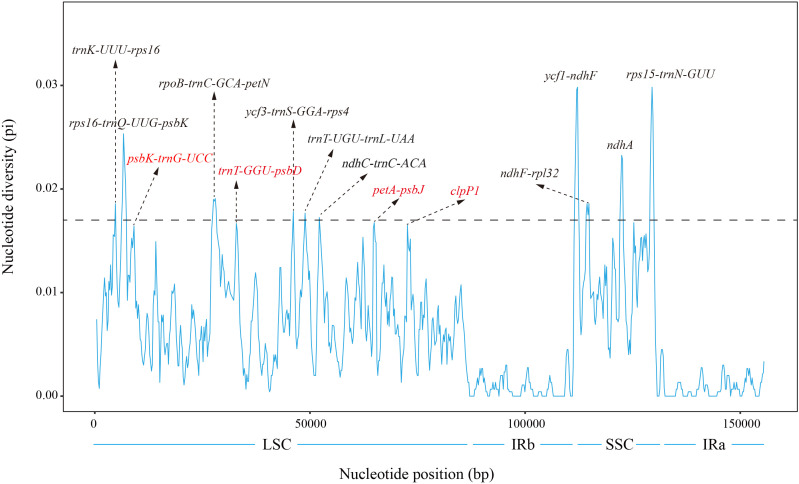
Nucleotide diversity (Pi) of the chloroplast genome of the six *Periploca* species from China. Sliding window length: 800 bp; step size: 200 bp. X-axis: position of the sliding window. Y-axis: nucleotide diversity of each window.

To further assess the sequence variation in the *Periploca* cp genomes, we calculated the nucleotide diversity value (Pi) of the cp genomes of the six *Periploca* species using the DnaSP software. The analysis indicated that Pi values ranged from 0 to 0.029 (*ycf1-ndhF*), with the IR regions having the lowest nucleotide diversity and the SSC region having the highest nucleotide diversity. By analyzing the calculated nucleotide diversity, ten hypervariable regions were detected, and most of them were located in the SSC and LSC regions (**Figure 7**). In addition, in the SSC region, the IGSs of *ndhF-rpl32*, *ycf1-ndhF*, and *rps15-trnN-GUU* showed high nucleotide diversity among the species, and the polymorphism rate of the *ndhA* gene was also high, with a Pi value greater than 0.017. In the LSC region, the highest diversity was found in the IGSs of *rps16-trnQ-UUG-psbK*, with a Pi value of 0.02515, Additionally, the IGSs of *trnK-UUU-rps16*, *rpoB-trnC-GCA-petN*, *ycf3-trnS-GGA-rps4*, *trnT-UGU-trnL-UAA*, and *ndhC-trnC-ACA* showed high nucleotide diversity among the species, with Pi values greater than 0.017.

### 3.5 Validation of potential molecular markers based on the chloroplast genome analysis

**Figure 8:**
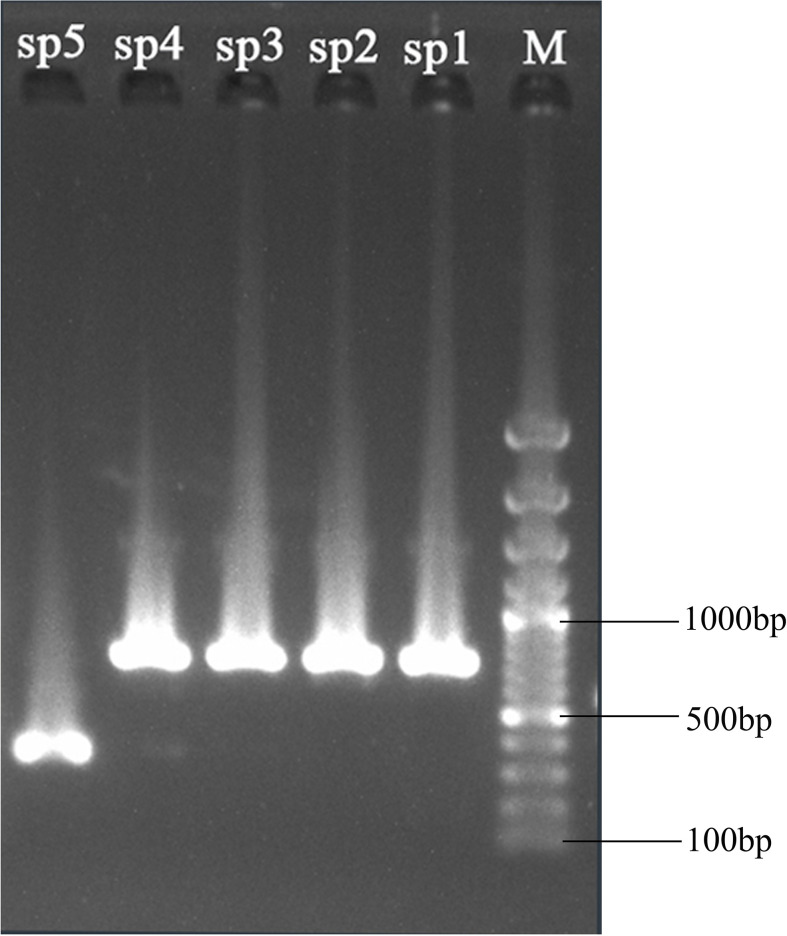
Gel electrophoresis of the amplification products using the designed primers (**Supplementary Table S7**, wzk10). Lane M is the standard DNA ladder. The lanes from right to left (marked sp1, sp2, sp3, sp4, and sp5) correspond to the products amplified from *P. forrestii*, *P. tsiangii*, *P. floribunda*, *P. calophylla*, and *P. chrysantha*, respectively.

**Figure 9:**
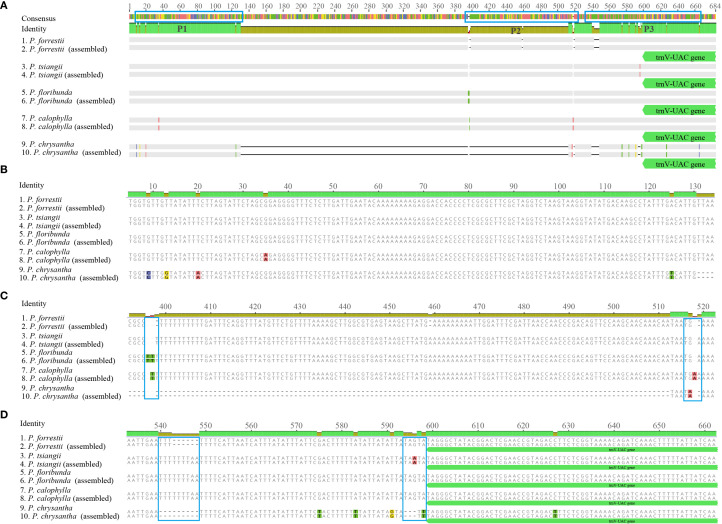
Sequence alignment of PCR products for the five *Periploca* species of the *ndhC-trnC-ACA* region. **(A)** Overall view of the alignment of the PCR products and assembly sequences; the blue frame indicates the area of variation. 1, 3, 5, 7, and 9 correspond to the sequences from the PCR products, and 2, 4, 6, 8, and 10 correspond to the assembled sequences. **(B–D)** show larger versions of the blue frames from left to right (marked P1, P2, and P3, respectively) in **(A)**.

According to the cp genome analysis, sixteen specific primer pairs were designed for the conserved sequences flanking the previously identified variable regions among the five sequenced *Periploca* species (**Supplementary Table S7**). The PCR products of these five species were sequenced to validate the hypervariable regions as potential species-specific molecular markers. The results demonstrated high consistency between Sanger sequencing and next-generation sequencing data in these hypervariable regions. Using the *ndhC-trnC-ACA* region as an example, the primer pair 10 successfully amplified target fragments in all five *Periploca* samples, with specific primers distinguishing *P. chrysantha* via PCR (**Figure 8**). Comparative analysis between high-throughput sequencing data and PCR product sequencing revealed indels across three intervals (P1, P2, and P3) in the *ndhC-trnC-ACA* region (**Figure 9A**). The chloroplast genome of *P. chrysantha* showed a 381 bp deletion in this region compared to the other four species, serving as a diagnostic marker (**Figure 8**). Additionally, in the P1 region, *P. chrysantha* exhibited four nucleotide mutations at distinct positions compared to the other species, while the *P. calophylla* was characterized by an A base at position 35 (**Figure 9B**). In the P2 region, all species except *P. floribunda* showed 1–3 bp deletions near position 400. Species-specific indels near position 520 further facilitated discrimination among the remaining four species (**Figure 9C**). The P3 region contained diagnostic deletions: *P. chrysantha* (9 bp), *P. forrestii* (6 bp), while the other three species showed no deletions. Furthermore, *P. tsiangii* could also be distinguished by a nucleotide mutation at position 596 (base A) (**Figure 9D**). Collectively, these variable regions effectively differentiated all five sequenced *Periploca* species, suggesting their utility as potential molecular markers for species identification within this genus.

### 3.6 Phylogenetic analysis

**Figure 10:**
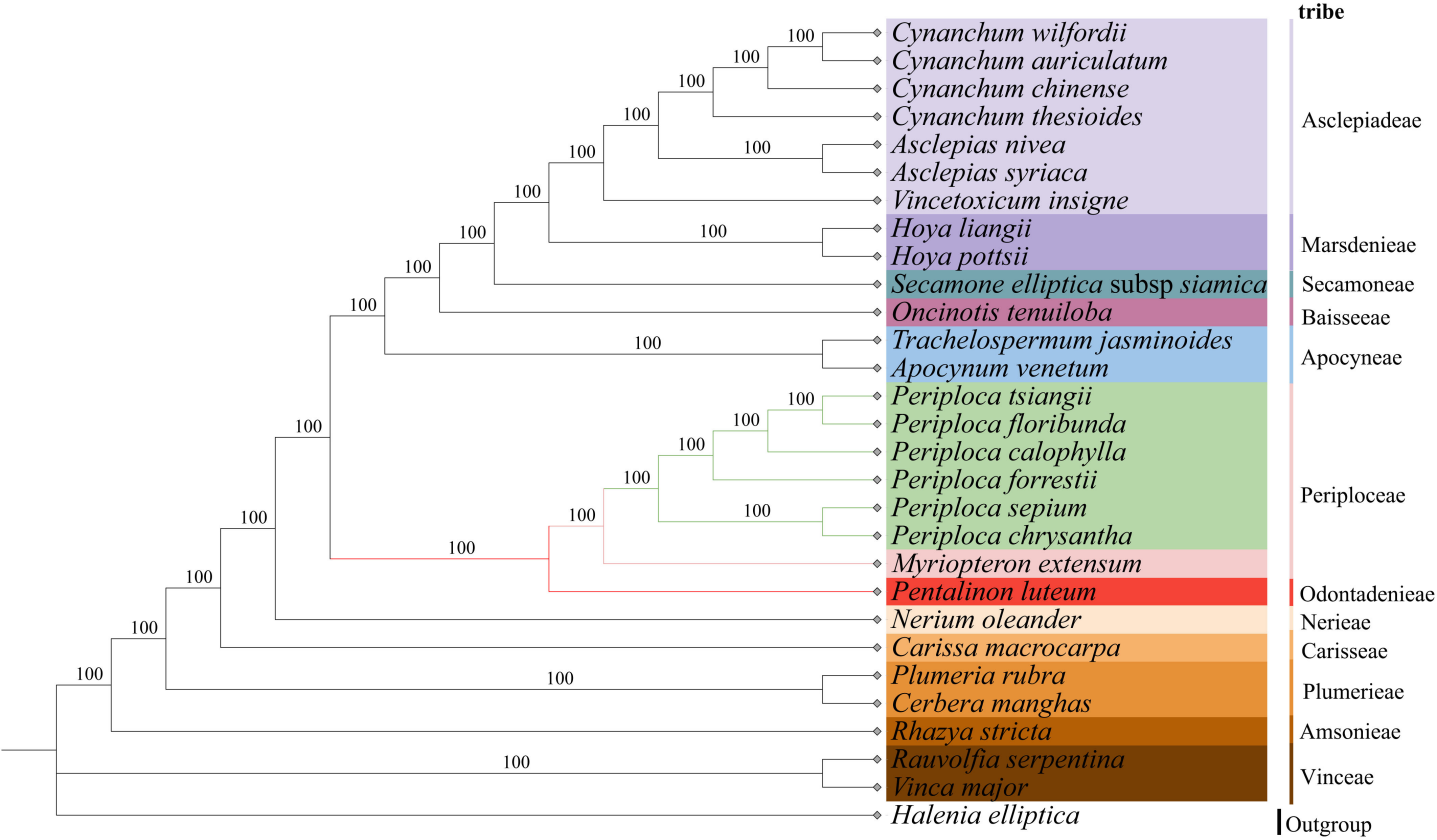
Phylogenetic tree of 29 species including six species of the *Periploca* genus in the Apocynaceae family from China based on complete chloroplast genomes obtained by the maximum likelihood method. The numbers above the branches represent bootstrap support value for ML methods. .

In this study, we used *Halenia elliptica* D. Don (Gentianaceae) as an outgroup. Based on complete cp genome sequences from 28 Apocynaceae species (including the six *Periploca* species from China), we employed maximum likelihood (ML) and Bayesian inference (BI) methods to infer phylogenetic relationships. The results revealed strong topology concordance between the two approaches, with all nodes exhibiting maximum support values (100% bootstrap (BS) support in ML) (**Figure 10**) and 1.0 Bayesian posterior probabilities (PP) in BI (**Supplementary Figure S2**). Given the topological congruence, only the ML tree is presented here for clarity. The 28 species in the two phylogenetic trees (excluding *H. elliptica*) were grouped into 12 major clades. Furthermore, in the Periploceae clade, the six *Periploca* species formed a strongly supported monophyletic group (BS = 100% at all nodes) together with *Myriopteron extensum*, which is consistent with traditional taxonomic classifications of tribe Periploceae. In this clade, the six *Periploca* species were resolved as a distinct subclade (100% BS support), demonstrating that they constitute a monophyletic lineage phylogenetically distinct from other genera in Periploceae. The six *Periploca* species from China were categorized into two distinct groups: one comprising *P. sepium* and *P. chrysantha*, and the other encompassing the remaining four species (*P. forrestii*, *P. calophylla*, *P. floribunda*, and *P. tsiangii*). These two groups’ classification aligns with the clustering of morphological characteristics and was consistent with the taxonomic results presented in both the *Flora Reipublicae Popularis Sinicae* and the *Flora of China*. Among the four species documented in the *Flora Reipublicae Popularis Sinicae* (*P. sepium*, *P. forrestii*, *P. calophylla*, and *P. floribunda*) and the five species (including *P. tsiangii*) recorded in *Flora of China*, *P. sepium* was distinguished from others by its membranous leaves. Notably, *P. chrysantha* was introduced as a new taxon (Yao et al., 2002) after these two monographs; morphologically, it shares traits with both membranous leaves and floral characteristics similar to *P. sepium*, which is consistent with the phylogenetic analyses in this study (**Supplementary Figures S3**, **S4**).

## 4 Discussion

### 4.1 The necessity of molecular delimitation in *Periploca*

Principal component analysis (PCA) of morphological traits revealed that the first two principal components collectively explained 92.0% of the total variance, with Dim1 (66.6%) significantly correlated with both leaf and floral traits (**Figure 2A**). This underscores the importance of floral morphology for accurate species identification, which aligns with the challenges in traditional taxonomy and highlights the necessity of supplementary molecular markers for reliable discrimination. Comparative analyses of complete cp genomes have proven effective in enhancing species resolution in many plant groups, making a thorough examination of cp genomic variations within *Periploca* a research priority.

### 4.2 Structural conservation and divergence in chloroplast genomes

The cp genomes of the six *Periploca* species were highly conserved in size, structure, gene content, and GC content, exhibiting a maximum length difference of only 627 bp. The overall GC content was stable (38.10–38.19%), but varied across different regions, with the IR regions exhibiting a higher GC content than the LSC and SSC regions. This is likely due to the presence of four rRNA genes (*rrn4.5*, *rrn5*, *rrn16*, and *rrn23*), which are duplicated in the IR region (Li et al., 2024). The uneven distribution of GC content across regions in *Periploca* cp genomes, as well as the trans-splicing events involving *rps12*, has also been reported in other plant species (Wang et al., 2024).

Long repeat sequences (≥ 30 bp) are ubiquitous in angiosperms and are considered to play an important role in genome stability and structural variation (Chen et al., 2022; Wu et al., 2024). Across the six studied species, a total of 60 long repeat sequences were identified, among which palindromic (P) and forward (F) repeats were the most common. Additionally, slight variations in the number and type of repeat units were observed among different species. Simple sequence repeats (SSRs), which are highly abundant and randomly distributed in genomes, serve as valuable molecular markers for studying population genetic relationships and phylogenetics (Provan et al., 2001; Li et al., 2024). In the cp genomes of the six *Periploca* species examined, the number of SSRs ranged from 55 to 73 per genome. Among these, mononucleotide A/T repeats dominated (66.67–84.51%), consistent with the AT bias typical of angiosperm cp genomes (Cavalier-Smith, 2002; Sablok et al., 2013, Sablok et al., 2013). These repeat elements and SSRs, particularly those in the more variable LSC region, serve as valuable potential molecular markers for phylogenetic and population genetic studies, and for the authentication of medicinal materials within this genus.

The contraction and expansion of the IR regions play a critical role in shaping the cp genome structure, influencing gene content, length, and organization through gene duplication, deletion, or pseudogenization (Song et al., 2022). Our comparative analysis of IR boundary shifts revealed dynamic expansion/contraction events among the six *Periploca* species, particularly affecting the *ycf1* gene. This structural variation, especially in the *ycf1-ndhF* intergenic spacer, underscores its potential as a DNA barcode for distinguishing closely related *Periploca* species.

### 4.3 Hypervariable regions as potential molecular markers

Divergent regions serve as effective molecular markers, providing valuable information for DNA barcoding and phylogenetic studies, as well as facilitating the identification of divergent hotspots for phylogenetic reconstruction (Wang et al., 2008; Antil et al., 2023). Our comparative genomic analysis (mVISTA) and nucleotide diversity (Pi) calculation confirmed that non-coding regions, especially intergenic spacers (IGS), were more variable than coding regions across the six cp genomes. We identified ten hypervariable regions (Pi > 0.017), primarily located in the LSC and SSC regions, which are promising for developing specific DNA barcodes. This inference was experimentally validated by designing primers targeting these regions and performing Sanger sequencing (**Figure 10**). The Sanger sequencing results were highly consistent with the NGS data, and species-specific indels and SNPs within these hypervariable regions effectively differentiated all five sequenced *Periploca* species, confirming their utility as molecular markers for the genus *Periploca*.

### 4.4 Phylogenetic relationships and taxonomic implications

Phylogenetic analysis is of great significance in clarifying the relationship between species and plays a key role in the protection, rational development, and utilization of plant resources (Liu et al., 2020). Chloroplast genomes are widely used to explore phylogenetic relationships at lower taxonomic levels because they can solve problems that morphological taxonomy cannot (Bonelli et al., 2024). In traditional classifications, the subfamily Periplocoideae is distinguished from other subfamilies by features such as tetrad pollen and free filaments (Jiang and Li, 1977). Within the subfamily Periplocoideae, the tribe Periploceae is differentiated from other tribes by its corona and filaments being simultaneously attached to the base of the corolla and fused with the filaments (Jiang and Li, 1977). Among the Periploceae, the the presence of heteromorphic corona lobes is a key characteristic of the genus *Periploca*, making it easily distinguishable from other genera.

In this study, the phylogenetic analyses based on complete cp genomes using ML and BI methods yielded congruent, highly supported trees. The six *Periploca* species formed a clade with *Myriopteron extensum* within the tribe Periploceae, consistent with traditional taxonomy. Within *Periploca*, two major clades were resolved: one comprising *P. sepium* and *P. chrysantha*, and the other containing *P. forrestii*, *P. calophylla*, *P. floribunda*, and *P. tsiangii*. This phylogenetic structure is largely congruent with the morphological clustering pattern revealed in this study (**Figure 2B**). The close relationship between *P. sepium* and *P. chrysantha* is supported by shared morphological traits (e.g., decussate leaf arrangement, membranous leaves, and thickened and reflexed corolla lobes). Thus, the phylogenetic reconstruction provides a robust framework for the taxonomic treatment and future utilization of these species.

## 5 Conclusion

The rapid advancement of high-throughput DNA sequencing technology and bioinformatics has significantly increased interest in the cp genomes for species identification and phylogenetic studies of medicinal plants, owing to their abundant genetic information and remarkable conservation (Liu et al., 2024). In this study, we sequenced, assembled, and annotated the cp genomes of five *Periploca* species from China, combined them with published cp genomes of *P. sepium* from NCBI, and comprehensively analyzed the cp genome sequences, structures, and features of the species for the first time. We screened out the differential sequences for identification of this genus, and explored the phylogenetic relationships among *Periploca* from China, as well as at the intra- and intergeneric levels. Our findings indicate that the cp genomes of *Periploca* can effectively resolve species-level identification and phylogenetic issues within the genus. This study serves as a valuable complement to conventional reliance on leaf morphology for identifying *Periploca* specimens lacking flowers or fruits, thereby facilitating the accurate medicinal application of plants in this genus.

## Data Availability

The datasets presented in this study can be found in online repositories. The names of the repository/repositories and accession number(s) can be found in the article/**Supplementary Material**.
