# Supplementary material for: Comparative chloroplast genomes and phylogenetic analysis of six Periploca species from China provide insights into the distinction of members of this small medicinal genus
Source: Front Plant Sci. 2025 Sep 22;16:1564539. doi: 10.3389/fpls.2025.1564539 (PMC12497752; doi:10.3389/fpls.2025.1564539)
Supplement: Supplementary file 1 [file DataSheet1.pdf]

1. Supplementary Material
Supplementary Tables: 7
Supplementary Figures: 4

1.1 Supplementary Tables
1.2 Supplementary Figures

1.1 Supplementary Tables

SUPPLEMENTARY TABLE S1 Morphological characters of *Periploca* from China used for morphometric analyses.

Symbol	Meaning	Encoding Information
LA	Leaf arrangement	Opposite and decussate (0), distichous opposite (1)
LT	Leaf texture	Leathery/sub-leathery (0), membranous (1)
LS	Leaf shape	Ovate-oblong/lanceolate (0), oblong/elliptic-lanceolate (1), narrowly lanceolate/linear (2)
LV	Leaf venation	Dense lateral veins (0), 20–25 lateral veins (1)
LL	Leaf length	Measured data
LW	Leaf width	Measured data
LR	Leaf length-to-width ratio	Measured data
FD	Flower diameter	Measured data
CF	Corolla morphology	Thickened and reflexed at middle (0), neither thickened nor reflexed (1)
CS	Corolla shape	Oblong (0), lanceolate-linear (1)
CL	Corolla length	Measured data
CW	Corolla width	Measured data
CR	Corolla length-to-width ratio	Measured data
PL	Paracorolla length	Measured data
PS	Paracorolla indumentum	Pubescent (0), glabrous (1)
CD	Calyx diameter	Measured data
CG	Calyx glands	5 glands (0), 10 glands (1)
SL	Stamen length	Measured data
AS	Anther shape	Ovate (0), auriculate-sagittate (1)

SUPPLEMENTARY TABLE S2 Morphological data matrix of six *Periploca* species from China.

**Taxa**	**Feature Encoding**																		
	LA	LT	LS	LV	LL	LW	LR	FD	CF	CS	CL	CW	CR	PL	PS	CD	CG	SL	AS
P. forrestii	1	0	2	0	6.08	0.97	6.52	1.19	1	0	0.65	0.25	2.6	0.48	0	0.38	0	0.15	0
P. tsiangii	1	0	2	0	5.3	1.13	5.05	3.07	1	1	1.58	0.23	6.87	0.55	0	0.29	0	0.15	1
P. floribunda	1	0	1	0	5.13	1.74	3.01	1.07	1	0	0.53	0.2	2.42	0.26	0	0.25	0	0.11	0
P. calophylla	1	0	1	0	5.23	1.83	2.92	1.06	1	0	0.47	0.22	2.44	0.27	0	0.27	0	0.12	0
P. chrysantha	0	1	0	1	7.4	2.83	2.69	2.2	0	0	1.14	0.52	3	0.84	1	0.58	1	0.38	0
P. sepium	0	1	0	1	6.6	2.15	3.14	1.97	0	0	1.12	0.49	2.33	0.89	1	0.61	1	0.29	0

SUPPLEMENTARY TABLE S3 GenBank accession number of chloroplast genomes of *Periploca* and other genera.

Subfamily	Species	GenBank
Asclepiadoideae	*Cynanchum wilfordii*	KT220733
	Cynanchum auriculatum	KT220734
	Cynanchum chinense	MW415427
	Cynanchum thesioides	MW864598
	Asclepias syriaca	KF386166
	Asclepias nivea	KF539844
	Vincetoxicum insigne	MH748558
	Hoya liangii	MH678666
	Hoya pottsii	MH678667
	Secamone elliptica subsp *siamica*	OP133598
Baisseoideae	*Oncinotis tenuiloba*	KJ953908
Apocynaceae	*Apocynum venetum*	MT365042
	Trachelospermum jasminoides	MK783315
	Pentalinon luteum	KJ953909
Periplocoideae	*Periploca forrestii*	MZ557568
	Periploca tsiangii	OP718510
	Periploca floribunda	PP153941
	Periploca calophylla	PP153940
	Periploca chrysantha	PP153942
	Periploca sepium	MH752592
	Myriopteron extensum	NC_079616
Nerioideae	*Nerium oleander*	KJ953907
Carissoideae	*Carissa macrocarpa*	KX364402
Allamandoideae	*Plumeria rubra*	MN812495
	Cerbera manghas	MT527963
Melodinoideae	*Rhazya stricta*	KJ123753
Rauvolfioideae	*Vinca major*	NC_065203
	Rauvolfia serpentina	MN746301
Gentianoideae	*Halenia elliptica*	MT228726

SUPPLEMENTARY TABLE S4 Genes annotated in the chloroplast genome of the six *Periploca* from China.

Category	Gene group	Gene name
Self-replication	Ribosomal RNA genes	*rrn4.5S*(×2)、*rrn5S*(×2)、*rrn16S*(×2)、*rrn23S*(×2)
	Transfer RNA genes	*trnA-UGC*[a](×2)、*trnC-ACA*[a]、*trnC-GCA*、*trnD-GUC*、*trnE-UUC*、*trnF-GAA*、*trnG-GCC*、*trnG-UCC*[a]、*trnH-GUG*、*trnI-GAU*[a](×2)、*trnK-UUU*[a]、*trnL-CAA*(×2)、*trnL-UAA*[a](×2)、*trnL-UAG*、*trnM-CAU*(×4)、*trnN-GUU*(×2)、*trnP-UGG*、*trnQ-UUG*、*trnR-ACG*(×2)、*trnR-UCU*、*trnS-GCU*、*trnS-GGA*、*trnS-UGA*、*trnT-GGU*、*trnT-UGU*、*trnV-GAC*(×2)、*trnV-UAC*[a]、*trnW-CCA*、*trnY-GUA*
	Small subunits of ribosomes	*rps2*、*rps3*、*rps4*、*rps7*(×2)、*rps8*、*rps11*、*rps12*[ac](×2)、*rps14*、*rps15*、*rps16*[a]、*rps18*、*rps19*
	Large subunits of ribosome	*rpl2*[a](×2)、*rpl14*、*rpl16*、*rpl20*、*rpl22*、*rpl23*(×2)、*rpl32*、*rpl33*、*rpl36*
	RNA polymerase subunits	*rpoA*、*rpoB*、*rpoC1*[a]、*rpoC2*
Genes for photosynthesis	NADH deoxygenase subunits	*ndhA*[a]、*ndhB*[a](×2)、*ndhC*、*ndhD*、*ndhE*、*ndhF*、*ndhG*、*ndhH*、*ndhI*、*ndhJ*、*ndhK*
	Photosynthetic system I subunits	*psaA*、*psaB*、*psaC*、*psaI*、*psaJ*
	Photosynthetic system II subunits	*psbA*、*psbB*、*psbC*、*psbD*、*psbE*、*psbF*、*psbH*、*psbI*、*psbJ*、*psbK*、*psbL*、*psbM*、*psbT*、*psbZ*
	photosystem I assembly factor	*ycf3*[b]、*ycf4*
	Cytochrome subunits	*petA*、*petB*[a]、*petD*[a]、*petG*、*petL*、*petN*
	ATP synthase subunits	*atpA*、*atpB*、*atpE*、*atpF*[a]、*atpH*、*atpI*
	Ribulose-1,5-bisphosphate carboxylase large subunit	*rbcL*
Gene for biosynthesis	Maturase	*matK*
Genes of unknown function	Protease	*clpP1*[b]
	Envelope membrane protein	*cemA*
	Acetyl-coenzyme A carboxylase subunit	*accD*
	C-type cytochrome synthesis gene	*ccsA*
	Translation initiation factor	*infA*
	Transcription factors	*pbf1*
	Conserved open reading frames	*ycf1*(×2)、*ycf2*(×2)、*ycf15*(×2)

Note: ‘a’gene containing one intron, ‘b’gene containing two introns, ×2 gene in the IR region with two copies present, ‘c’ trans-spliced gene.

SUPPLEMENTARY TABLE S5 Types and amounts of simple sequence repeats (SSRs) in the chloroplast genomes of the six *Periploca* species from China.

SSR Type	*P. forrestii*		*P. tsiangii*		*P. floribunda*		*P. calophylla*		*P. chrysantha*		*P. sepium*	
	Number	Ration(%)	Number	Ration(%)	Number	Ration(%)	Number	Ration(%)	Number	Ration(%)	Number	Ration(%)
mono-	43	76.8	39	68.4	39	70.9	47	78.3	63	86.3	62	87.3
di-	1	1.8	2	3.5	2	3.6	2	3.3	2	2.7	1	1.4
tri-	4	7.1	8	14.0	6	10.9	4	6.7	3	4.1	3	4.2
tetra-	5	8.9	4	7.0	4	7.3	4	6.7	5	6.8	5	7.0
penta-	3	5.4	4	7.0	4	7.3	3	5.0	0	0.0	0	0.0
Total	56	100	57	100	55	100	60	100	73	100	71	100

SUPPLEMENTARY TABLE S6 Simple sequence repeats (SSRs) among six *Periploca* species chloroplast genomes.

Taxa	SSR	Frequency
P. forrestii	AATCT/AGATT	1
P. forrestii	AATAT/ATATT	1
P. forrestii	AAAAT/ATTTT	1
P. forrestii	AATT/AATT	2
P. forrestii	AATG/ATTC	1
P. forrestii	AAAT/ATTT	1
P. forrestii	AAAG/CTTT	1
P. forrestii	AAT/ATT	3
P. forrestii	AAG/CTT	1
P. forrestii	AT/AT	1
P. forrestii	C/G	
P. forrestii	A/T	43
P. tsiangii	AATCT/AGATT	1
P. tsiangii	AATAT/ATATT	2
P. tsiangii	AAAAT/ATTTT	1
P. tsiangii	AATT/AATT	2
P. tsiangii	AATG/ATTC	1
P. tsiangii	AAAT/ATTT	1
P. tsiangii	AAAG/CTTT	
P. tsiangii	AAT/ATT	7
P. tsiangii	AAG/CTT	1
P. tsiangii	AT/AT	2
P. tsiangii	C/G	1
P. tsiangii	A/T	38
P. floribunda	AATCT/AGATT	1
P. floribunda	AATAT/ATATT	2
P. floribunda	AAAAT/ATTTT	1
P. floribunda	AATT/AATT	2
P. floribunda	AATG/ATTC	1
P. floribunda	AAAT/ATTT	1
P. floribunda	AAAG/CTTT	
P. floribunda	AAT/ATT	5
P. floribunda	AAG/CTT	1
P. floribunda	AT/AT	2
P. floribunda	C/G	1
P. floribunda	A/T	38
P. calophylla	AATCT/AGATT	1
P. calophylla	AATAT/ATATT	1
P. calophylla	AAAAT/ATTTT	1
P. calophylla	AATT/AATT	2
P. calophylla	AATG/ATTC	1
P. calophylla	AAAT/ATTT	1
P. calophylla	AAAG/CTTT	
P. calophylla	AAT/ATT	3
P. calophylla	AAG/CTT	1
P. calophylla	AT/AT	2
P. calophylla	C/G	
P. calophylla	A/T	47
P. chrysantha	AATCT/AGATT	
P. chrysantha	AATAT/ATATT	
P. chrysantha	AAAAT/ATTTT	
P. chrysantha	AATT/AATT	1
P. chrysantha	AATG/ATTC	1
P. chrysantha	AAAT/ATTT	3
P. chrysantha	AAAG/CTTT	
P. chrysantha	AAT/ATT	2
P. chrysantha	AAG/CTT	1
P. chrysantha	AT/AT	2
P. chrysantha	C/G	2
P. chrysantha	A/T	61
P. sepium	AATCT/AGATT	
P. sepium	AATAT/ATATT	
P. sepium	AAAAT/ATTTT	
P. sepium	AATT/AATT	1
P. sepium	AATG/ATTC	1
P. sepium	AAAT/ATTT	3
P. sepium	AAAG/CTTT	
P. sepium	AAT/ATT	2
P. sepium	AAG/CTT	1
P. sepium	AT/AT	1
P. sepium	C/G	2
P. sepium	A/T	60

SUPPLEMENTARY TABLE S7Primers used for PCR amplification in this study.

Primer name	Sequence (5'-3')	Start position	End position	Tm (°C)	Length (bp)	Names of the intervals
wzk01_F	GATCAGTCGCGGTCTTCCAA	4,089	4,108	50	871	*trnK-UUU-rps16*
wzk01_R	GGGGGAGTGGTTTGTATATAACTT	4,937	4,960			
wzk02_F	ATGTCCTTCAAGTCGCACGT	6,030	6,049	50	665	*rps16-trnQ-UUG-psbK*
wzk02_R	TCGCTCGATGTATTTTCTTTGAAT	6,672	6,695			
wzk03_F	AGCGAGATTCAAAGAAAATACATCGA	6,666	6,691	56	747	*rps16-trnQ-UUG-psbK*
wzk03_R	CGAATCCTTCCGTCCCAGAG	7,394	7,413			
wzk04_F	TCCATCCCCGAGCATTTTGA	27,128	27,147	55	810	*rpoB-trnC-GCA-petN*
wzk04_R	ATGTTGCCTATACGTAATTCTCAGA	27,914	27,938			
wzk05_F	AGCACTTAACACACCCCTTT	27,609	27,628	55	773	*rpoB-trnC-GCA-petN*
wzk05_R	GCCATGCCGCCAAAATGATA	28,363	28,382			
wzk06_F	AAATGCCTCCCTTTCTCCCG	45,446	45,465	55	1,025	*ycf3-trnS-GGA-rps4*
wzk06_R	GGTTCGGACAATTTTCTTCCCTT	46,449	46,471			
wzk07_F	AGCGGGCTCACATAACATCA	47,572	47,591	55	788	*trnT-UGU-trnL-UAA*
wzk07_R	TGTCGTTCCCCTTTTCTTTCA	48,340	48,360			
wzk08_F	GGGGAACGACATCATAACGC	48,350	48,369	55	1,038	*trnT-UGU-trnL-UAA*
wzk08_R	TGTGCCAGGAACCAGATTTGA	49,368	49,388			
wzk09_F	CGGGGCTAAAACTCCGGAAA	51,650	51,669	55	589	*ndhC-trnC-ACA*
wzk09_R	GCCAGTCTCTTGCTTGTTCT	52,220	52,239			
wzk10_F	AGAACAAGCAAGAGACTGGC	52,220	52,239	55	742	*ndhC-trnC-ACA*
wzk10_R	GCATGTTGGGTCTTTGAAACAG	52,941	52,962			
wzk11_F	GGCAGAATACCGTCACCCAT	111,249	111,268	55	737	*ycf1-ndhF*
wzk11_R	AGCTATCAAATATGTAGGGGGCG	111,964	111,986			
wzk12_F	CCGCCCCCTACATATTTGAT	111,963	111,982	55	717	*ycf1-ndhF*
wzk12_R	CTCTATGGGGTAAGGGAATTTCA	112,658	112,680			
wzk13_F	ACGGGAAGTGGGATGAAAGG	114,057	114,076	55	877	*ndhF-rpl32*
wzk13_R	ACGCTGTCCAATATCCCTTCC	114,914	114,934			
wzk14_F	TCATCTCATACGGCTCCTCAA	122,050	122,070	56	836	*ndhA*
wzk14_R	GCAGTAGAGACCGCTTACCC	122,867	122,886			
wzk15_F	TCGCCGAAAGTCCGATTGTT	129,021	129,040	55	729	*rps15-trnN-GUU*
wzk15_R	AGGGAATAAGACAAGCAAGGAA	129,729	129,750			
wzk16_F	CACAATCGAGTTCTTGTTTCCAG	129,655	129,677	54	751	*rps15-trnN-GUU*
wzk16_R	GGCAGAATACCGTCACCCAT	130,387	130,406			

1.2 Supplementary Figures

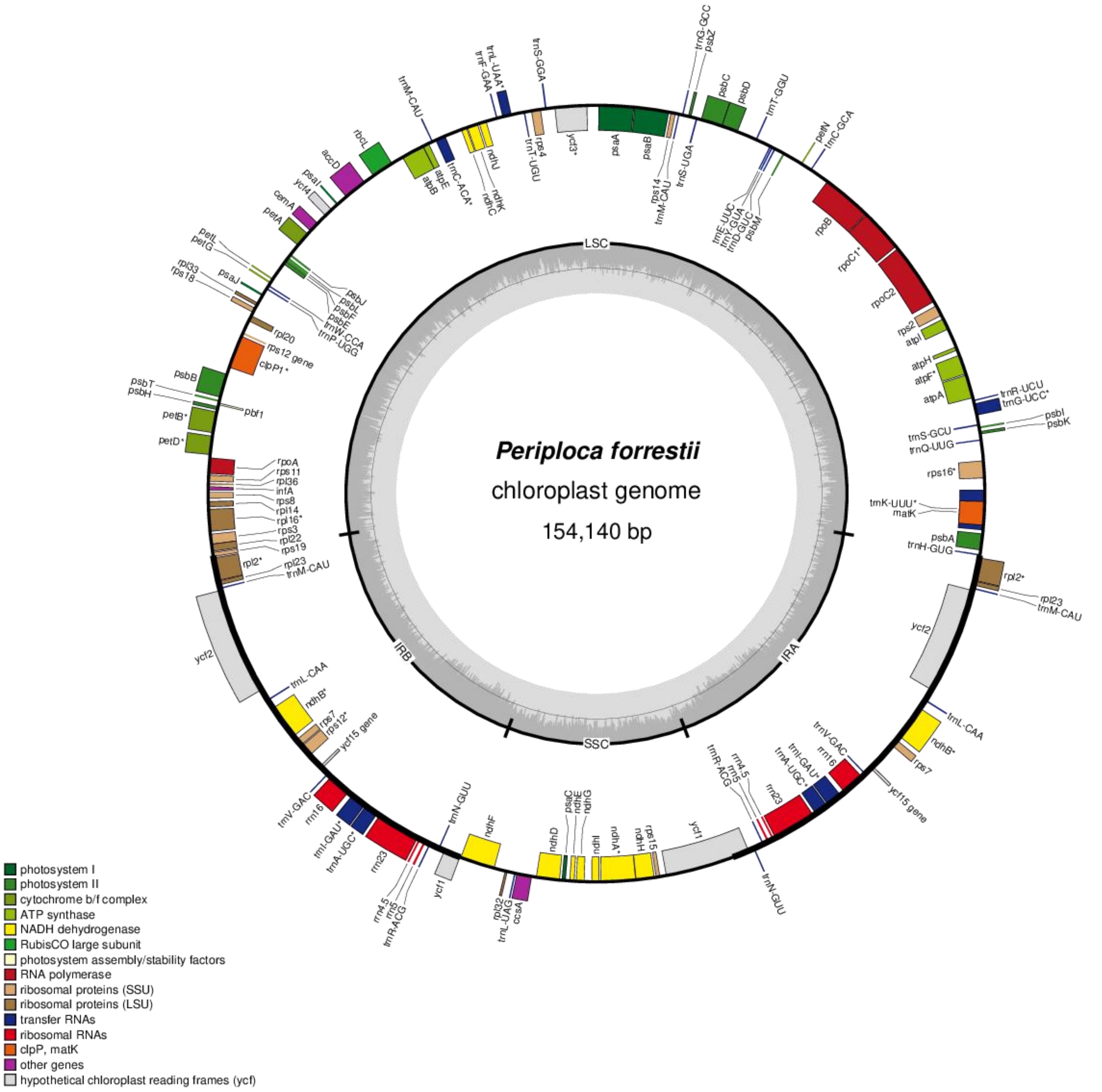

SUPPLEMENTARY FIGURE S1

Chloroplast genome map of *P. forrestii*. Genes inside the large circle are transcribed clockwise, while those outside are transcribed counter-clockwise. The genes are color-coded according to their functional annotations.

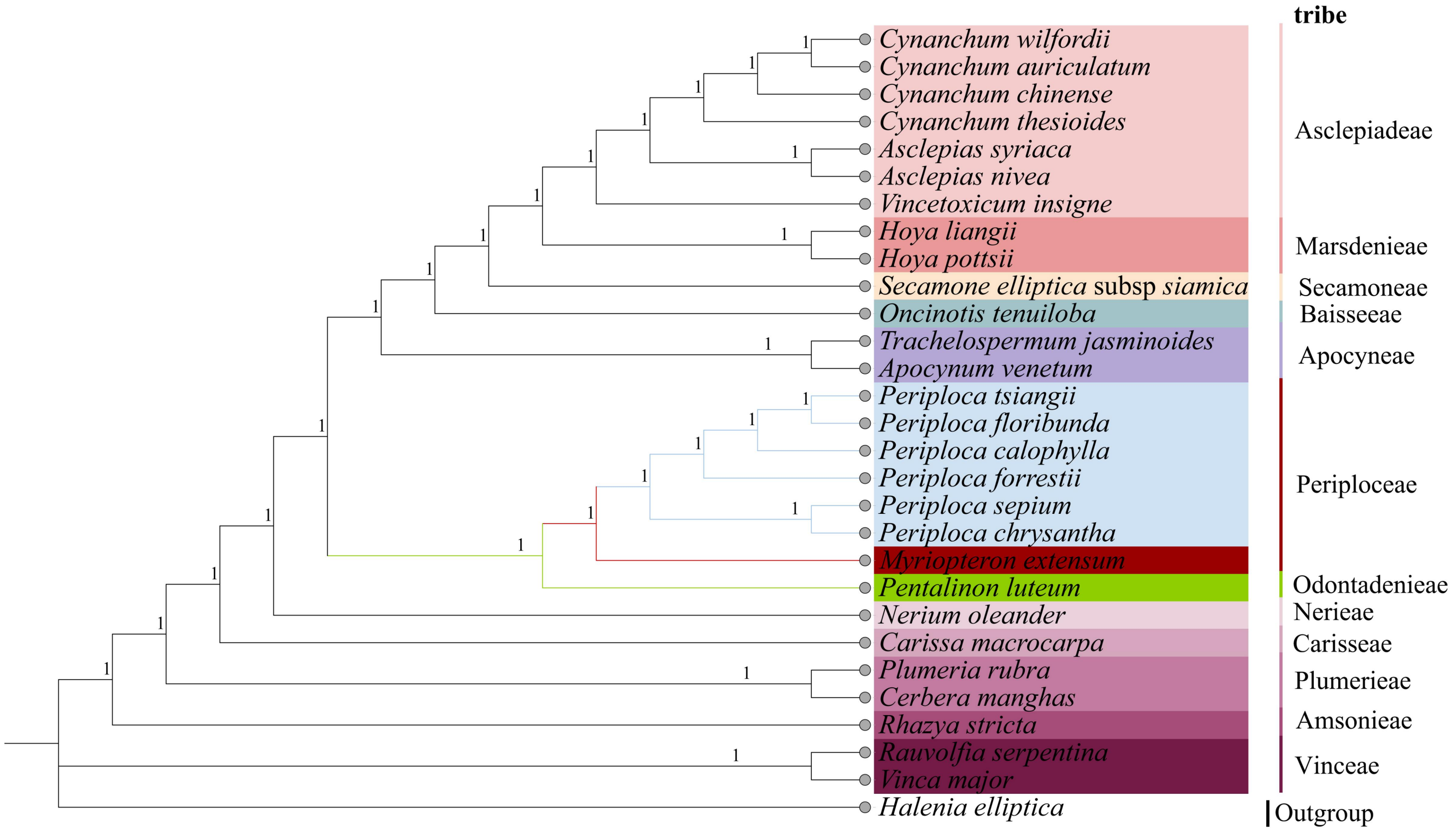

SUPPLEMENTARY FIGURE S2

Phylogenetic tree of 29 species including six species of the *Periploca* genus in the Apocynaceae family from China based on complete chloroplast genomes obtained by the Bayesian inference method.

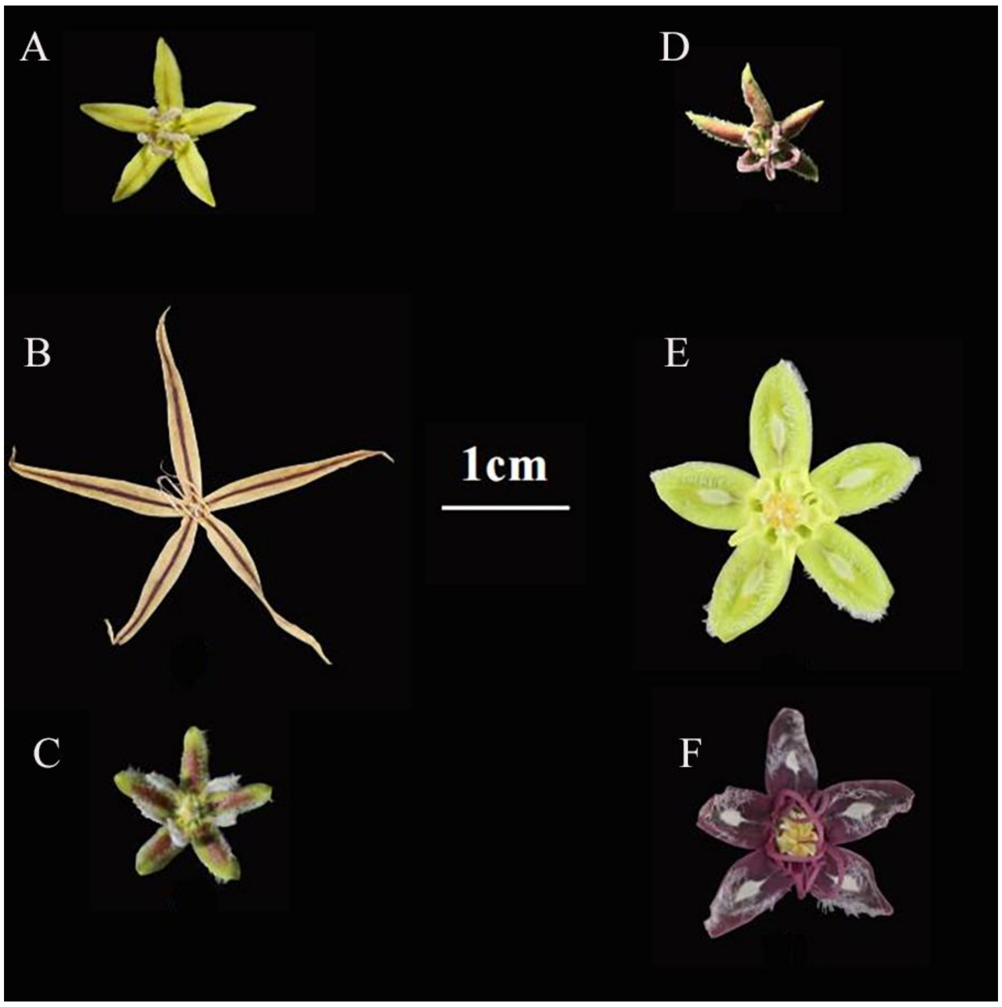

SUPPLEMENTARY FIGURE S3
Flower morphology of the six *Periploca* species from China. **(A)** *P. forrestii*. **(B)** *P. tsiangii*. **(C)** *P. floribunda*. **(D)** *P. calophylla*. **(E)** *P. chrysantha*. **(F)** *P. sepium*.

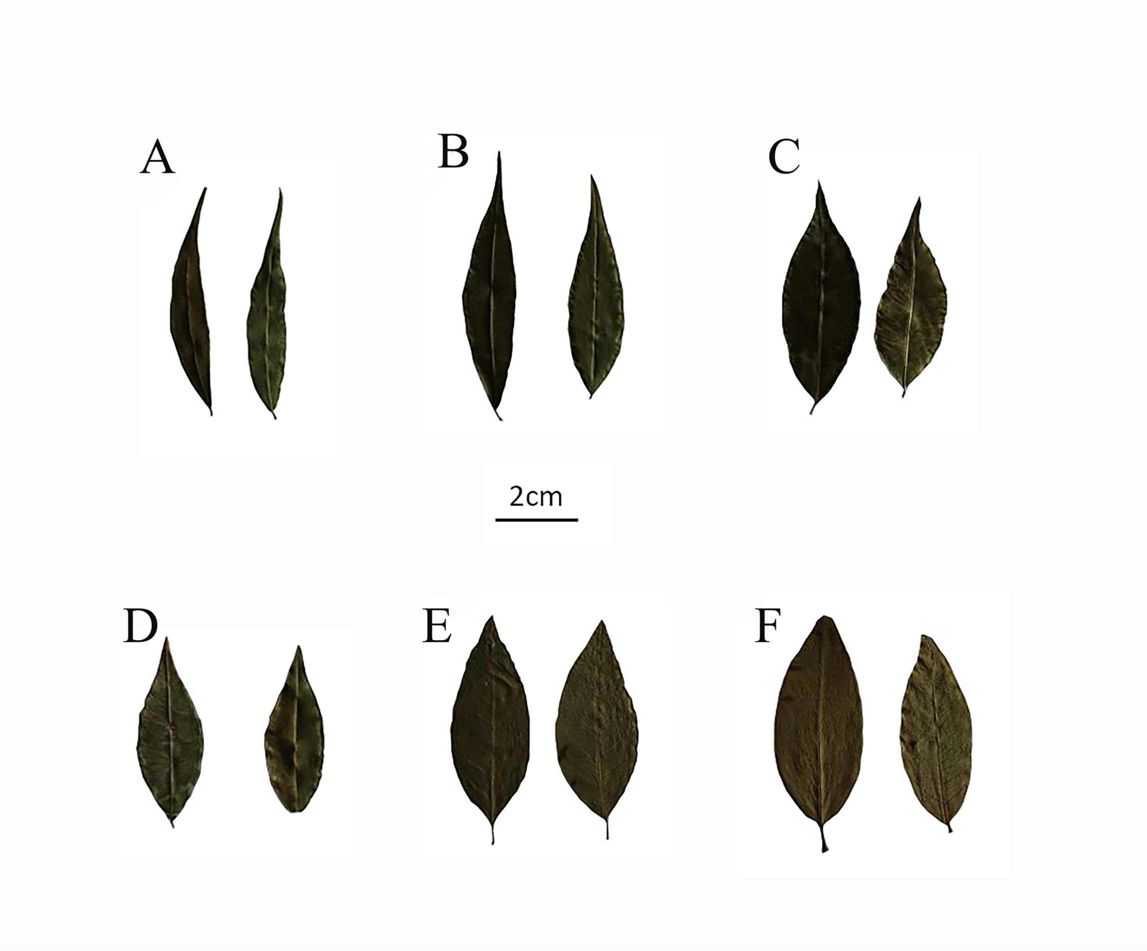

SUPPLEMENTARY FIGURE S4
Leaf morphology of the six *Periploca* species from China. **(A)** *P. forrestii*. **(B)** *P. tsiangii*. **(C)** *P. floribunda*. **(D)** *P. calophylla*. **(E)** *P. chrysantha*. **(F)** *P. sepium*.
